# The effect of fornix deep brain stimulation in brain diseases

**DOI:** 10.1007/s00018-020-03456-4

**Published:** 2020-01-23

**Authors:** Huajie Liu, Yasin Temel, Jackson Boonstra, Sarah Hescham

**Affiliations:** 1 Department of Neurosurgery, Maastricht University Medical Center, PO Box 5800, 6202 AZ Maastricht, The Netherlands; 2 European Graduate School of Neuroscience (EURON), Maastricht University, Maastricht, The Netherlands

**Keywords:** Deep brain stimulation, Fornix, Alzheimer disease, Rett syndrome, Traumatic brain injury, Temporal lobe epilepsy, Mechanisms

## Abstract

Deep brain stimulation is used to alleviate symptoms of neurological and psychiatric disorders including Parkinson’s disease, epilepsy, and obsessive–compulsive-disorder. Electrically stimulating limbic structures has been of great interest, and in particular, the region of the fornix. We conducted a systematic search for studies that reported clinical and preclinical outcomes of deep brain stimulation within the fornix up to July 2019. We identified 13 studies (7 clinical, 6 preclinical) that examined the effects of fornix stimulation in Alzheimer’s disease (*n* = 9), traumatic brain injury (*n* = 2), Rett syndrome (*n* = 1), and temporal lobe epilepsy (*n* = 1). Overall, fornix stimulation can lead to decreased rates of cognitive decline (in humans), enhanced memory (in humans and animals), visuo-spatial memorization (in humans and animals), and improving verbal recollection (in humans). While the exact mechanisms of action are not completely understood, studies suggest fornix DBS to be involved with increased functional connectivity and neurotransmitter levels, as well as enhanced neuroplasticity.

## Introduction

Deep brain stimulation (DBS) within the basal-ganglia network has become a safe routine second-tier therapy in patients with Parkinson’s disease, essential tremor, and dystonia [1, 2], whereas its application to other neural pathways such as the circuit of Papez is under active investigation. The circuit of Papez is considered one of the major pathways of the limbic system and is primarily involved in emotional expression, neurovegetative function, and memory [3]. While DBS applied to limbic targets has been evaluated for patients with treatment-resistant depression [4–6] and obsessive–compulsive disorder [7], recently studies have begun to explore the applicability of DBS in a widening array of psychiatric conditions including Alzheimer’s disease (AD) dementia [8, 9]. The classical circuit of Papez consists of the hippocampal formation, fornix, mammillary bodies, mammillothalamic tract, anterior thalamic nucleus, cingulum, and the entorhinal cortex [10]. Damage to structures within the circuit of Papez can result in anterograde amnesia in patients, i.e., an inability to create new episodic memories [11–15]. In line with this, damage to the fornix in experimental animals and humans is known to cause memory deficits [16–19]. In the current review we focus on the effects of fornix DBS on brain diseases, discuss advances within DBS systems and the potential mechanisms of action underlying symptom reduction, and briefly describe preclinical and clinical studies with regard to AD, Rett syndrome, traumatic brain injury, and temporal lobe epilepsy to elucidate their potential within future research. Lastly, we highlight the use of fornix DBS to restore memory loss and discuss overall considerations.

## Methods

For this review, we searched PubMed for clinical and preclinical studies in English literature with the search terms “deep brain stimulation”, “fornix”, “Alzheimer disease”, “Rett syndrome”, “dementia”, “traumatic brain injury”, and “temporal lobe epilepsy”. Key words were used independently and in different combinations. Relevant articles were also chosen from review papers, original research articles, and book chapters. Studies describing fornix DBS in patients and rodents were included.

Clinical outcomes were Mini-Mental State Examination (MMSE), Alzheimer’s Disease Assessment Scale–Cognitive subscale (ADAS-cog), Rey Auditory-Verbal Learning Test (RAVLT), visual-spatial memory via the Medical College of Georgia Complex Figure Test, visual confrontational naming via the Boston Naming Test Short Form (BNT) and Free and Cued Selective Reminding Test. Outcome measures for preclinical studies were performance in behavioral tests (e.g., Morris water maze and fear conditioning). Articles aimed to study the effects of DBS in target areas other than the fornix were excluded. Moreover, case reports and articles written in languages other than English were excluded. We considered all relevant studies published until July 2019 in the present review, which in total amounted to 13 (7 human and 6 rodent studies).

## What is DBS?

Deep brain stimulation is a minimally invasive surgical method in which stimulation electrodes are stereotactically implanted into specific brain targets. The implantation of DBS electrodes can be performed under local or general anaesthesia. The most commonly used DBS system uses a multi-contact stimulating electrode that is connected with an internal pulse generator through a subcutaneous wire. The DBS device and the settings can be accessed externally with a wireless connected controller. Stimulation parameters can be adjusted to obtain the best possible therapeutic effects with little or no side effects. Different stimulation parameters such as frequency, amplitude, pulse width, the choice of bipolar or monopolar stimulation, and continuous or intermittent stimulation can be adjusted. Some DBS systems also allow for steering, meaning that a specific part of the circular contact can be activated or de-activated. Severe adverse effects related to the surgical procedure are intracerebral haemorrhages that occur in 1–2% of patients while less severe or reversible events such as infections, lead, and pulse generator problems occur in a vast minority of the patients [20].

## Advances in DBS technology

Although DBS is an established treatment for many neurological disorders such as Parkinson’s disease, tremor, epilepsy, and dystonia, there are still limitations in terms of efficacy, side effects, and battery consumption. To accommodate these limitations, advances in DBS technology have focused on stimulation procedures, electrodes, and pulse generator design.

With regard to limited efficacy and the occurrence of side effects, researchers found that these challenges may be due to modulating not only pathological but also physiological neural activity [21, 22]. For this reason, adaptive DBS (aDBS) where stimulation is only applied when necessary might be advantageous. In aDBS, a device records local field potential activity (or other physiological signals) from the implanted DBS electrode and delivers simultaneous stimulation through the same electrode based on the recorded signal. The recorded physiological signals can then be fed back to dynamically alter and optimize stimulation parameters [23]. Clinical implementation of aDBS has been limited due to a range of challenges in optimizing each component of the feedback [24] but the approach promises substantial benefits in the future.

Another refinement for DBS is called coordinated reset (CR) DBS which aims towards therapeutic reshaping of neuronal connectivity by harnessing synaptic plasticity (e.g., spike timing-dependent plasticity) [25, 26]. In this method, brief high-frequency pulse trains are given through the different contacts of the stimulation electrode in treatment blocks for a few consecutive days resulting in the disruption of pathologically synchronized oscillations. The goal of CR-DBS is to decrease synaptic weights thereby debilitating pathological connectivity and synchrony [27]. In a non-human primate model of parkinsonism, CR-DBS of the subthalamic nucleus (STN) for 5 consecutive days resulted in acute motor improvements and, in contrast to traditional DBS, showed benefits persisting up to 2 weeks after stimulation [28].

The advent of directional leads is another technological advancement in DBS that allows targeting to be made more accurately with the goal of avoiding side effects [29]. Unlike conventional DBS leads which use cylindrical electrodes, directional leads are comprised of radially segmented electrodes that allow the stimulation field to be moved in the plane perpendicular to the lead, or shaped using anodes and cathodes to steer stimulation in a particular direction [30]. Given the novelty of this approach, however, there is currently no firm clinical evidence.

Finally, the use of rechargeable implantable pulse generators (rIPG) pretense another innovation in the field and have been proven effective and applicable in Parkinson’s disease, essential tremor and dystonia [31]. These rlPGs have a longevity of at least 15 years in contrast to the non-rechargeable IPGs showing a mean longevity of 3–5 years. The major advantage is that patients need fewer replacement surgeries while a disadvantage is that patients must charge the rIPGs a few times a week [32].

## Mechanisms of DBS

Initial hypotheses about the mechanism of DBS were based on observed similarities between DBS and lesion therapy on the alleviation of symptoms in Parkinson’s disease. For example, internal globus pallidus (GPi) DBS [33–35] and pallidotomy [36] both produce similar effects on parkinsonian motor symptoms. Thus, DBS was initially believed to generate a depolarization block of neurons around the stimulating electrode [37, 38]. Later, it was shown that DBS might also have an effect on neuronal firing patterns. These changes in firing patterns are thought to prevent transmissions of pathologic bursts and oscillatory activity resulting in the reduction of disease symptoms through compensatory processing of sensorimotor information.

In addition to the local electrical effects of DBS, researchers found that DBS could also induce neurochemical changes locally and through the stimulated network. For instance, DBS of the anterior thalamus for the treatment of epilepsy in a rodent model induces the release of hippocampal adenosine [39]. Moreover, DBS has shown to induce plastic changes with regard to synaptic plasticity and neurogenesis. In line with this, Gondard et al. [40] have shown that acute fornix DBS can modulate neurotrophic factors such as brain-derived neurotrophic factor (BDNF) and vascular endothelial growth factor (VEGF) as well as synaptic plasticity markers such as growth associated protein 43, α-synuclein and synaptophysin. Hippocampal neurogenesis has additionally been induced following thalamic DBS in a group of adult rats [41]. The authors concluded that an involvement of the Papez circuitry is necessary in mediating the effects of DBS and in the treatment of cognitive and behavioral disorders.

## The anatomy, connections, and functions of the fornix

**Fig. 1:**
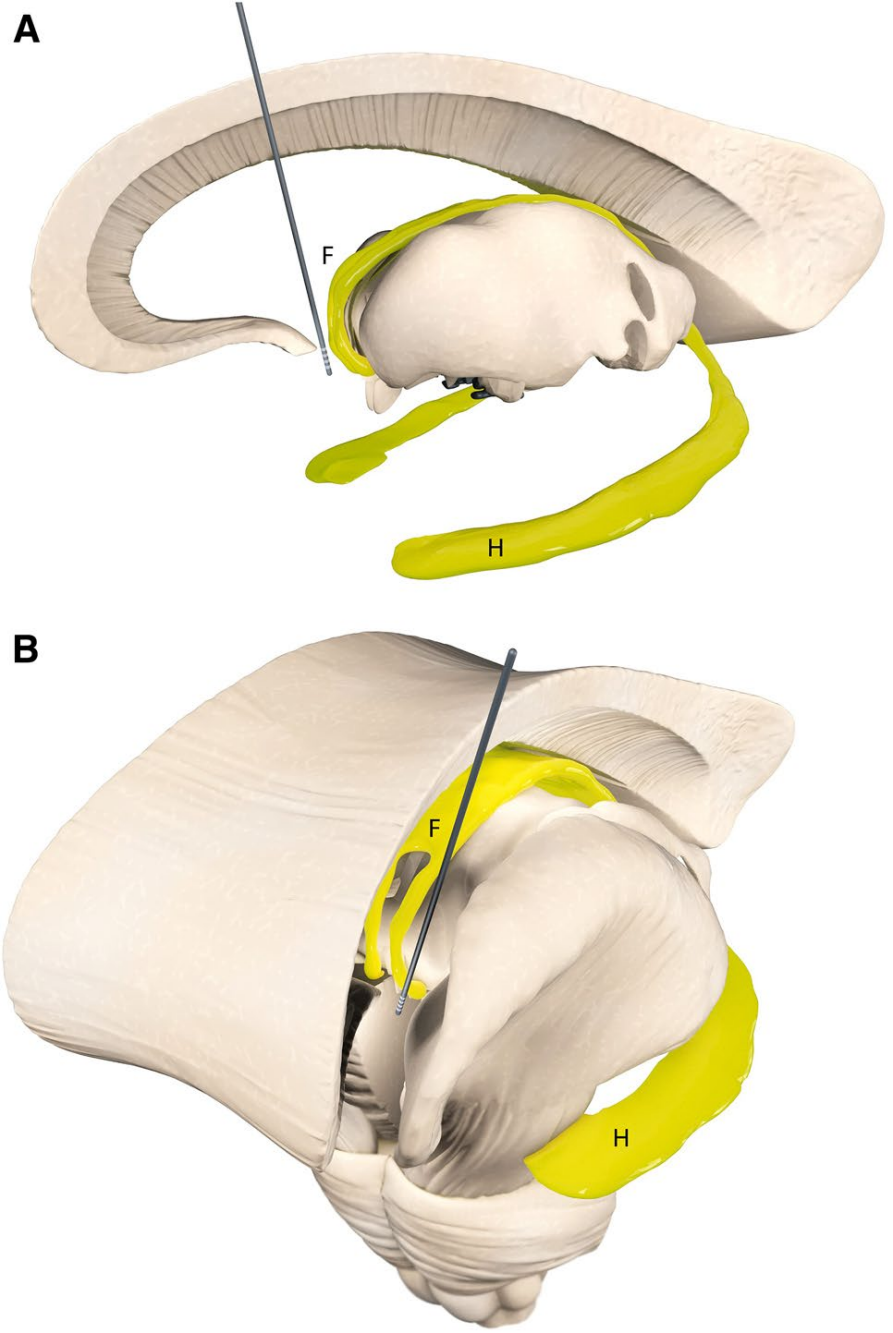
Simplified illustration of anatomical targeting for fornix deep brain stimulation in clinical studies. The fornix (F) and the hippocampus (H) are depicted in yellow. Efferent fibers of the hippocampus known as the alveus join together to form the fimbria. Beneath the splenium of the corpus callosum, the fimbria separates from the hippocampus and becomes the crus of the fornix. The left and right crura then converge to form the body of the fornix. The body of the fornix travels anteriorly and divides again near the anterior commissure. The left and right parts separate into the anterior pillars, and there is also an anterior/posterior divergence. The posterior fibers (called the postcommissural fornix) of each side continue through the hypothalamus to the mammillary bodies. The anterior fibers (precommissural fornix) end at the septal nuclei and nucleus accumbens of each hemisphere. **a** Sagittal view of fornix DBS electrode location. **b** Frontal view of fornix DBS electrode location in one hemisphere

The fornix arises from output fibers of the hippocampus located in the medial temporal lobe below the base of the lateral ventricle. Under the ependymal surface of the lateral ventricle is a thin layer of efferent fibers known as the alveus that mainly ascend from the pyramidal cells of the hippocampus and form a fringe of fibers known as the fimbria. Beneath the splenium of the corpus callosum the white matter of the fimbria separates from the hippocampus and becomes the crus of the fornix [42, 43]. Sometimes the fimbria and fornix are referred to as the fimbria–fornix complex to highlight its functional unity and anatomic connections. The left and right crura then converge in the medial plane beneath the trunk of the corpus callosum to form the body of the fornix. The lateral portions of the body of the fornix are joined by a thin triangular lamina that contain some commissural fibers that connect the two hippocampi known as commissure of the fornix or commissure of the hippocampus. The body of the fornix travels anteriorly and divides again near the anterior commissure. The left and right parts separate into the anterior pillars, and there is also an anterior/posterior divergence. The posterior fibers (called the post-commissural fornix) of each side continue through the hypothalamus to the mammillary bodies and then to the anterior nuclei of thalamus which project to the cingulate cortex. The anterior fibers (pre-commissural fornix) end at the septal nuclei and nucleus accumbens of each hemisphere. An anatomic illustration of the fornix can be found in Fig. 1.

The most common types of neuroglia cells in the fornix are oligodendrocytes, followed by astrocytes, and microglial cells [44]. The primary function of these neuroglia cells is to form myelin, maintain homeostasis, and provide support and protection for neurons amongst others. Neuroanatomical and axonal tract tracing studies reveal that fibers in the fimbria–fornix fall into two categories, thin unmyelinated and thick myelinated [45]. In particular, it was shown that a major source of cholinergic innervation of the hippocampus comes from the medial septum via the fimbria–fornix pathway and contains axons that are unmyelinated or thinly myelinated [46]. GABAergic septohippocampal axons also project to the hippocampus via the fimbria–fornix pathway and contain thickly myelinated fibers [47]. The cholinergic neurons synapse onto all hippocampal cell types while the GABAergic neurons terminate on hippocampal GABAergic neurons [46].

The fornix is an integral part of the classical Papez circuit. When considering the rodent and primate Papez circuits, the core connections of the hippocampal-diencephalic-cingulate network are, respectively, homologous. One of the major differences is in the connections of the cingulate cortices in rodents and primates (for review see [48]). The fornix is imperative to the function of formation and consolidation of memory in rodents and primates [49, 50] as it has been shown that lesions of the fornix lead to various amnestic syndromes [51].

## Studies on fornix DBS

We identified 13 studies that examined the effects of fornix DBS in Alzheimer disease (*n* = 9), traumatic brain injury (*n* = 2), Rett syndrome (*n* = 1), and temporal lobe epilepsy (*n* = 1). A summary of these studies can be found in Table 1. In the following, we will review each disorder separately.

**Table 1:** A summary of fornix DBS studies presented in this review

Subject	Type of stimulation	Memory task	Effect	References
Human (patients with TBI) *N* = 4	Continuous DBS using a burst pattern (200 Hz in 100 ms trains, 5 trains/s, 100 µs pulse width, 7 mA, bipolar and bilateral stimulation)	Rey auditory verbal learning test, Medical College of Georgia Complex Figure Test, Boston Naming Test	Burst stimulation of the fornix was associated with a robust reversible improvement in immediate and delayed performance on the Medical College of Georgia Complex Figure Test	Miller et al. [58]
Rats (model of TBI) *N* = 21	Low-frequency (5 Hz), high-frequency (130 Hz), and theta-burst stimulation (200 Hz in 50 ms trains, five trains per second; 60 µA biphasic pulses)	Swim T‐Maze, Morris Water Maze	Deficits in learning and memory after TBI are improved following DBS of the fornix	Sweet et al. [60]
Human (patients with intractable epilepsy) *N* = 11	Bilateral, high amplitude, low pulse width, low frequency (8 V, 0.2 μs, 5 Hz), for 4 h	MMSE	An increase of MMSE scores during stimulation. Hippocampal spikes were significantly reduced during and outlasting each stimulation session. Seizure odds (*n* = 7) were reduced by 92% in the 2 days that followed stimulation	Koubeissi et al. [63]
Mice (model of RTT) *N* = 21	Bilateral, 130 Hz, 60 µs pulse duration, for 1 h per day for 14 days	Fear conditioning, Morris water maze, open field, light–dark box, wire hang and dowel walk, accelerating rotarod, three-chamber interaction, and pain threshold	Forniceal DBS in RTT mice rescues contextual fear memory as well as spatial learning and memory	Hao et al. [65]
Human (morbid obesity patient) *N* = 1	Bilateral, 3–5 V, 130 Hz and 60 μs pulse width, continuous for 3 weeks	Neuropsychological tests, e.g., verbal learning test, WAIS attention index, spatial associative learning, etc	Significant improvements on the California Verbal Learning Test and Spatial Associative Learning Test	Hamani et al. [50]
Human (AD patients) *N* = 6	Bilateral, 3.0–3.5 V, 130 Hz, and 90 μs pulse width, continuous for 12 months	ADAS-cog, MMSE	Possible improvements and/or slowing in the rate of cognitive decline at 6 and 12 months in some patients	Laxton et al. [72]
Human (AD Patients) *N* = *6*	Bilateral, 3.0 V, 130 Hz and 90 μs pulse width, continuous for 12 months	ADAS-cog, MMSE	Local volume increase in parahippocampal gyri, right superior temporal gyrus, left parietal lobule and bilateral precuneus as well as thalamus and superior frontal gyrus	Sankar et al. [73]
Human (AD patient) *N* = 1	Bilateral, 2.5 V, 130 Hz and 210 ms pulse width, continuous for 12 month	ADAS-cog, MMSE, Free and Cued Selective Reminding Test	Cognitive scores worsened after 6 months but returned to baseline after 12 months of chronic DBS	Fontaine et al. [74]
Human (patients with mild AD) *N* = 42	Bilateral, 3.0–3.5 V, 130 Hz, with a pulse width of 90 microseconds at the top, or second from top, of the 4 electrode contacts, continuous for 12 month	ADAS-Cog-13, CDR-SB, California Verbal Learning Test-Second Edition (CVLT-II), the Alzheimer’s Disease Cooperative Study Activities of Daily Living scale (ACDS-ADL)	Participants aged ≥ 65 years show less cognitive decline while there was possible worsening in patients below 65 years with stimulation	Lozano et al. [75]
Rats (model of experimental dementia) *N* = 10	Bilateral, 100 and 200 μA, 10 and 100 Hz, 100 μs pulse width, acute stimulation	OLT	Memory enhancement in high current densities (frequency-independent)	Hescham et al. [78]
Rats *N* = 29	Bilateral, 100 Hz, 100 µA and 100 µs pulse width for 1 h	N/A	Fornix DBS induced a selective activation of cells in the CA1 and CA3 subfields of the dorsal hippocampus, a substantial increase in the levels of extracellular hippocampal acetylcholine	Hescham et al. [79]
Rats (transgenic rat model of AD, tgF344) *N* = 10	Permanent, bilateral, and unipolar stimulation (130 Hz, 80 µs, 100 µA)	N/A	Amyloidosis, inflammatory responses, and neuronal loss decreased in both cortex and hippocampus after DBS in the fornix	Leplus et al. [76]
Mice (transgenic mouse model of AD, 3xTg) *N* = 50	Monophasic, 1 h (100 Hz, 100 μs pulses, 100 μA)	Morris water maze	Fornix DBS improved learning and long- term memory after 3 and 6 weeks with significant differences driven mostly by males	Gallino et al. [77]

The table shows the subjects involved, the type of stimulation, memory task, and the behavioral outcome of the surgical intervention

*ADAS-cog* Alzheimer's Disease Assessment Scale-cognitive subscale, *CDR-SB* Clinical Dementia Rating Scale Sum of Boxes, *OLT* object location task, *DBS* deep brain stimulation, *MMSE* mini-mental state examination, *WAIS* Wechsler Adult Intelligence Scale

### Traumatic brain injury (TBI)

Traumatic brain injury (TBI) is one of the world’s most devastating causes of morbidity and mortality. TBI affects more than 1.5 million patients in Europe and 1.7 million people in the United States every year. TBI is considered to be an injury to the head which is related to symptoms or signs caused by injury, i.e., skull fracture, amnesia, decreased or altered levels of consciousness, neurological or neuropsychological abnormalities, or intracranial lesions [52].

Many TBI patients experience significant functional deficits, e.g., somatic disorders (such as headaches or dizziness), emotional sickness (such as sleep disturbance, anxiety, or depression), impaired executive function, and memory loss [53]. Based on past TBI studies memory dysfunction is common and results from abnormal hippocampal activity [54]. Memory abnormalities caused by TBI are most likely to have a complicated underlying mechanism involving synaptic dysfunction, cell death, changes in hippocampal connectivity, and neural pathway dysfunction. While hippocampal theta oscillations may be associated with learning and memory, especially in spatial memory [55, 56], it is important to note that hippocampal theta oscillations have been reported to be decreased after TBI [57].

In a recent study, theta burst stimulation of the dorsal fornix was reported to induce memory improvement in patients with TBI [58]. Because of this, it was hypothesized that the modulation of neural activity via the hippocampus by fornix DBS may improve cognitive recovery after TBI. Stimulation electrodes were thus implanted in the proximal fornix and dorsal hippocampal commissures of four TBI patients. Three patients received their electrode on their language dominant side and one patient received it on their non-dominant side. A diffuse evoked potential was generated by the electrode in the head and body of the ipsilateral hippocampus.

Memory tests were performed once a day for at least two consecutive days with different test forms each day such as verbal memory via Rey Auditory-Verbal Learning Test (RAVLT), visual-spatial memory via the Medical College of Georgia Complex Figure Test, and visual confrontational naming via the Boston Naming Test Short Form (BNT). All fornix electrodes were continuously stimulated using a burst pattern (200 Hz in 100 ms trains, 5 trains/s, 100 µs pulse width, 7 mA). Results showed that the burst stimulation of the fornix was correlated with an improvement in the Medical College of Georgia Complex Figure Test. It was hypothesized that the stimulation on the language-dominant side may improve verbal memory while on the non-dominant side it may improve visual memory. However, results showed that the stimulation of either side improved visual-spatial memory and reflects the role that both sides of the hippocampus have in spatial memory, especially in spatial relationships [59]. Since the sample size is very low, however, these results should be viewed as preliminary. The effect of fornix DBS on other functions, such as verbal memory and naming, did not produce any significant differences and appears to be much more complex, with considerable variability among patients after stimulation. It might be possible that the burst stimulation paradigm even has negative effects on some types of function, but this speculation needs to be further investigated.

Recently, different parameters of fornix stimulation in how they affect cognitively demanding tasks after TBI were investigated in male rats. Researchers implanted electrodes into the fornix and separated rats into a fluid-percussion injury group and a sham-operated group. A 60-s delayed non-match-to-sample (DNMS) swim T-maze was serially performed using four stimulation parameters: no stimulation (no stim), low frequency (5 Hz), high frequency (130 Hz), and theta-burst stimulation (TBS, 200 Hz in 50 ms trains, five trains per second; 60 mA biphasic pulses). In the cognitively demanding DNMS swim T-maze and a water maze there was a significant difference in performance between TBI + no stim and TBI + TBS groups but no significant difference between sham + no stim and TBI + TBS. The TBI + TBS group performed significantly more platform crossings in the probe trial and exhibited improved search strategy starting on day 3 when compared to TBI + no stim, demonstrating that fornix DBS with TBS improved memory after TBI. While there are limitations in this study, such as the low sample size and the stimulation settings being different from previous human studies, these results indicate that the modification of neural activity in the hippocampus induced by fornix TBS may constitute a new therapeutic method for memory deficits after TBI [60].

### Temporal lobe epilepsy (TLE)

Temporal lobe epilepsy (TLE) is the most common form of intractable epilepsy. The prevalence of TLE in developed countries ranges from 4 to 10 cases per 1000 [61]. Mesial TLE usually arises in the hippocampus, an area of the brain known for its involvement in memory. The efficacy and safety of DBS for epilepsy has been demonstrated by the SANTE trial where the anterior nucleus of the thalamus (ANT) was targeted [62]. Based on this trial, the U.S. Food and Drug Administration granted approval for DBS therapy for epilepsy. Although ANT-DBS was able to produce beneficial effects on seizure frequency, complaints of memory impairment occurred in 27% of patients over the course of the trial. For this reason, researchers have investigated whether the fornix can be used as alternative DBS target [63]. In one study, two epileptic patients were implanted with electrodes in the fornix, and nine were implanted with electrodes anterolateral to the splenium of the corpus callosum where the crus of the fornix has fibers that travel to the dorsal hippocampal commissure (the fornodorsocommissural tract). Low-frequency stimulation (bilateral, 5 Hz, 8 mA, 0.2 ms pulse width) in the fornix was given in 4 h blocks while a video-electroencephalography (EEG) unit was monitored simultaneously. Results indicated that the hourly Mini-Mental State Examination (MMSE) scores trended to increase during the stimulation period compared to pre-stimulation period, suggesting substantial memory improvement. Hippocampal spikes were additionally decreased in and after each low-frequency stimulation, and seizure odds (*n* = 7) were reduced by 92% in 2 days after the stimulation. Nevertheless, authors do not exclude a possible interference with anti-epileptic drugs for their spike and seizure analysis results. The half-life of the majority of anti-epileptic drugs is about 2 days and might have therefore confounded the seizure results even of subjects who received them > 2 days before fornix DBS. The study design also lacked an originally planned control with sham stimulation sessions, because the visible hippocampal responses on the raw EEG prevented blinding the EEG reader. Moreover, patients were only stimulated for 4 h/day for a total of 9 days (4 subjects underwent only 1 session, 5 subjects underwent 3 sessions, 1 patient had 2 sessions, and one patients underwent 9 sessions). This short‐term nature of stimulation, makes it difficult to make definitive conclusions about the chronic effects of fornix stimulation as it would be applied in patients.

### Rett syndrome (RTT)

Rett syndrome (RTT) is a progressive neurodevelopmental disorder caused by a loss of functional mutations in the methyl-CpG-binding protein 2 (MECP2) gene [64]. The main clinical symptoms include developmental deterioration of movement, loss of language and coordination skills, stereotypical hand movements, and microcephaly. Recently, it has been reported that high-frequency fornix stimulation in a RTT mouse model could improve cognitive deficits related to the dysfunction via regulating neural circuits involved in memory and learning development [65].

This research is the first application of potential therapeutic methods of a childhood intellectual disability disorder in a mouse model. Researchers implanted electrodes in the fimbria–fornix in female MeCP2+/− (RTT) and wild type (WT) mice. After biphasic fornix DBS (130 Hz, and 60 µs pulse width 1 h per day for 2 weeks) mice were subjected to behavioral tests including fear conditioning, water maze, open field, light–dark box, wire hang, dowel walk, accelerating rotarod, three-chamber interaction, and pain threshold. Results indicated that fornix DBS significantly improved spatial learning and spatial memory as well as contextual fear memory in WT and RTT mice but did not enhance locomotion, anxiety, pain threshold, motor learning, coordination, social behaviour, or body weight in RTT mice. Moreover, results showed that fornix DBS increased hippocampal neurogenesis and synaptic plasticity, which could improve learning and memory functions [66, 67].

### Alzheimer’s disease (AD)

More than 40 million people in the world have Alzheimer’s disease (AD). AD is a neurodegenerative disorder characterized by various pathological processes including regionally specific and sequential brain atrophy, amyloid plaques, neurofibrillary tangles, synaptic dysfunction, and neuronal cell death [68]. Patients suffer from progressive memory impairment and dementia leading to the worsening of everyday life [69]. So far, there are no clear effective treatments available to slow down the progression of AD. Equally, pharmacological therapeutic methods only alleviate symptoms temporarily and are not effective for all patients [70, 71].

In 2008 when a patient underwent DBS to treat obesity, the treatment did not have an influence on the patient’s appetite, but uniquely evoked a “déjà vu” experience leading to the hypothesis that bilateral stimulation of the fornix may help to improve memory [15]. Following this study, a Phase I research trial of bilateral fornix DBS was conducted in six mild to moderate AD patients and no sham control group. Bilateral stimulation of the fornix proved to be feasible and safe, having no serious adverse events [72]. The principle outcomes were that 4 out of 6 patients showed an improvement in their Alzheimer's Disease Assessment Scale-Cognitive Subscale (ADAS-Cog) scores 6 months after surgery, and 5 out of 6 patients showed a reduced decline in their Mini Mental State Examination (MMSE) one year after surgery. Moreover, a sustained partial reversal of hypometabolism was observed. It was shown in structural magnetic resonance imaging (MRI) that fornix DBS not only decreased the mean hippocampal atrophy but also increased the hippocampal volume in 2 patients 1 year after treatment, indicating the possibility for long-term structural plasticity driven by fornix DBS [73].

An additional prospective study was conducted to assess the safety and feasibility of fornix DBS in mild AD patients. During a 1 year study, recently diagnosed AD patients (*n* = 110) with predominant impairment of episodic memory were recruited, but only 8.2% of patients (*n* = 9) achieved all the criteria for inclusion and in the end only one patient accepted to be operated upon and accomplished the study [74]. Using permanent stimulation (bipolar, 130 Hz frequency, 210 ms pulse width, 2.5 V) in the fornix for 1 year, the patient was measured via memory test scores (ADAS-Cog, MMSE, FCSRT (Free and Cued Selective Reminding Test)), and compared to their baselines. Results showed that the memory test scores were stabilized and the mesial temporal lobes metabolism increased. This study suggested that fornix DBS is feasible, safe, and could act through antidromic stimulation of the hippocampus, even though only one AD patient was involved [74].

Because of the promising preliminary results, researchers embarked on a Phase II study of a yearlong, randomized, double-blind trial of fornix DBS in 42 mild AD patients. During the stimulation of the fornix, patients exhibited increased metabolism at 6 months but not at 12 months. Unexpectedly, patients < 65 years old (*n* = 12) trended to be worse with DBS ON versus OFF while patients ≥ 65 (*n* = 30) with DBS ON demonstrated not only increased cerebral glucose metabolism but also a trend towards beneficial clinical outcomes [75]. The authors concluded that this interaction in age and treatment might indicate that younger patients have a tendency towards a more malignant course of the disease. Another conclusion of this trial was that the stimulation parameters applied to AD patients were not disease-specific [75] and, retrospectively, the trial can be viewed as pre-mature. Evidently, a major drawback of current neuromodulation approaches is that the clinical application of DBS is moving faster than the scientific evidence supporting or discouraging its application. New indications, such as AD need to be backed up by robust scientific evidence to apply optimized protocols to the patients.

In a preclinical study that was the first to report about chronic fornix DBS in a transgenic rat model of Alzheimer’s disease, the effects of chronic fornix stimulation on amyloid burden, inflammation, and neuronal loss were investigated [76]. Researchers applied permanent, bilateral, and unipolar stimulation (130 Hz, 80 µs, 100 µA) 10 days after implantation surgery [76]. Results showed that amyloidosis, inflammatory responses, and neuronal loss decreased in both cortex and hippocampus after DBS in the fornix.

Recently, Gallino et al. designed an experiment of fornix DBS in an Alzheimer’s mouse model [77]. It combined brain imaging and behaviour by a proof-of-concept methodology in longitudinal assessments. After 1 h fornix DBS at 100 Hz, 100 μs pulse width and 100 μA, mice were assessed in the water maze. DBS treatment improved learning and long-term memory 3 and 6 weeks later, with significant differences driven mostly by males. Females tended to perform well regardless of stimulation status. Significant, persistent, volumetric changes were seen in diverse brains structures, such as the bilateral cingulate cortex areas where stimulation induced higher final volumes in males and lower final volumes in females. In contrast, the fimbria, alveus and external capsule displayed the opposite relationship, in which stimulation resulted in higher final volumes for females, and lower volumes for males. The greatest volumetric changes were found in the colliculi. These changes were unexpected, as these areas are not part the circuit of Papez and are associated with visual and auditory processing, respectively. It is possible that differences in visual/auditory processing and coordinated movements could affect the latencies to reach the target in the Morris water maze. The pronounced sex differences underscore the importance of conducting trials with both sexes. It is very often the case that females are excluded from preclinical experiments due to concerns that female hormone cycles will introduce variance. Conducting experiments with only male animals, however, can lead to false conclusions about the effectiveness, safety, and significantly limit generalizability of treatments under investigation in preclinical trials.

In another study, researchers applied bilateral fornix DBS with different stimulation parameters in a scopolamine-induced rat model of dementia. Scopolamine is a muscarinic acetylcholine receptor antagonist that mimics memory deficits. After being tested in different behaviour paradigms at different frequencies (10 and 100 Hz), different amplitudes (50, 100 and 200 μA), and with 100 μs pulse widths, it was found that fornix DBS improved spatial memory deficits and had no side-effects on anxiety and general motor activity [78].

Researches then performed c-Fos immunohistochemistry in the hippocampus as well as microdialysis sampling to investigate the neural mechanisms of fornix DBS in association with the memory improvement. It was found that fornix DBS selectively activated cells in the CA1 and CA3 sub-region of the hippocampus. Moreover, extracellular neurotransmitters such as acetylcholine in the hippocampus substantially increased 20 min after the stimulation while hippocampal glutamate levels were not significantly different compared to the baseline [79]. Interestingly, the release of acetylcholine was substantial when DBS was initiated with clear-cut behavioral effects, but declined over time despite ongoing DBS. However, the authors investigated these extracellular neurotransmitters with only the stimulation paradigm of 100 Hz, 100 μA and 100 μs. In continuing this research, it will be crucial to see whether an optimal release of acetylcholine could be achieved through different stimulation parameters of the fornix and lead to long-term therapeutic effects.

## Modulating memory loss with DBS

Effectively any neurological, neurodegenerative, toxic, or traumatic damage to brain structures within the circuit of Papez, especially the hippocampus, may lead to deficits in episodic memory that may resemble or precede AD. This holds true particularly in the absence of other neurological or neuropsychological symptoms or signs indicative of an alternative cause. The diagnostic procedure of memory impairment is based on a comprehensive clinical investigation (comprised of detailed medical histories, neurological, and psychiatric examination, etc.). Additional investigations to support the diagnosis of AD include biomarkers such as reduced Aβ_42_, increased tau in the cerebrospinal fluid, typical patterns in ^18^F-FDG-PET, and disproportionate atrophy involving medial, basal, and lateral temporal lobes and medial and lateral parietal cortices. Besides neuroanatomical alterations, synaptic degeneration, cell loss, neurotrophic failure, cellular genetics, and neuronal selective vulnerability are evident [80]. Circuit-wide neurochemical and electrophysiological changes also occur in AD, such as acetylcholine depletion [81] and abnormal alpha and theta rhythms [82]. Furthermore, neuroinflammation has been suggested to play a central role in the pathogenesis of AD [83]. In the course of the disease, microglia and astrocytes start to produce cytokines and pro-inflammatory mediators leading to chronic inflammation, the long-lasting and intense activation of which is thought to cause further neurodegeneration [83]. It is apparent that the pathophysiology of AD is complex and multifaceted. Some aspects like the initial causes of the disease, the abnormal formation of Aβ plaques, the mechanisms by which it affects neurons, the relation between the disruption of cholinergic pathways, and the cognitive deficits of AD are to date not fully understood.

Clinical and preclinical DBS studies targeting the fornix have shown to counteract some of the aforementioned pathological features. The phase I and phase II trials of fornix DBS for AD have indicated that fornix DBS is a feasible and safe methodology in AD patients, displaying inspiring early results for cognitive improvement. Moreover, fornix DBS can reverse some of the temporoparietal hypometabolism seen in AD [72].

In preclinical studies, it has been shown that DBS of the fornix improves impaired spatial memory and enhances neuronal activities in the hippocampus. In line with this, bilateral fornix DBS in the rat for 1 h induced expression of c-Fos, an immediate‐early marker of neural activation, in the hippocampus [79]. High-frequency fornix DBS was found to enhance levels of synaptophysin, a synaptic marker, in the hippocampus of normal rats [40]. BDNF and VEGF were also significantly increased 2.5 h after stimulation, suggesting that neurotrophic and proliferating factors are associated with electrical stimulation [40]. Chronic fornix DBS was performed in transgenic AD rats and showed Aβ_42_ plaque clearance in the cortex and hippocampus [76]. Moreover, it decreased astrogliosis and microglial activation and partly rescued neuronal loss in both cortex and the hippocampus. Another study has indicated that fornix DBS can lead to enhanced acetylcholine levels in the hippocampus [79].

To summarize, DBS has been found to exert beneficial effects in neuropathological hallmarks, molecular expression, and behavior in AD. So far, whether the effects on these biochemical markers will continue to improve with DBS until they reach a stable plateau or whether these markers will show natural fluctuations under various stimulation parameters, is not well understood and warrants further investigation.

## Discussion

The fornix composes an important afferent and efferent pathway from the hippocampus and medial temporal lobe. It contributes a direct afferent source from the hippocampus to the anterior thalamic nucleus. In the current review, we discussed the use of fornix DBS across several different neuropsychiatric disorders that are largely heterogeneous (TBI, TLE, RTT, and AD). In these studies, authors hypothesized that targeting the fornix with DBS can successfully alleviate cognitive deficits stemming from damaged brain structures within the circuit of Papez (e.g., the hippocampus).

In this regard, burst pattern fornix DBS was able to improve visual-spatial memory cognitive deficits in four TBI patients, but not verbal memory or naming. Besides memory loss, some TBI patients also experience somatic symptoms, behavioral changes, and neurological symptoms (such as dystonia, tremor). While fornix DBS might be able to alleviate cognitive impairment, other symptoms are less likely to be mitigated and would require additional treatment.

In TLE, stimulation of fiber bundles in structures such as the fornix can alter the threshold of seizure induction without affecting memory. Since only 11 patients were tested in this first trial, a new clinical trial involving 20 patients is currently underway sponsored by the George Washington University. It would be interesting to see if the authors can confirm their initial results.

For the treatment of memory loss in AD, DBS studies have exposed two regions of interest: the fornix and the nucleus basalis of Meynert (NBM). The NBM has wide cholinergic projections to the neocortex and some to the hippocampus. When applying DBS to these structures we are able to enhance memory in animals as well as in humans. It has been hypothesized that the beneficial memory effects following NBM-DBS are due to neuroprotective properties (for review see [84]). The current hypothesis for the fornix states that this effect is accomplished by driving fornix activity, both orthodromically as well as antidromically. This is supported by the view that large myelinated axons produce excitatory responses upon electrical stimulation [85]. Electrically stimulating the fornix proves to be effective in decreasing rates of cognitive decline [72, 74], enhancing memory [15], aiding visuo-spatial memory [86], improving verbal recollection [15], reducing Aβ_42_-related plaques and neuroinflammation [76], decreasing astrogliosis and migroglia levels [76], and increasing metabolism [72, 73].

A recent phase II trial of fornix DBS in 42 mild AD patients, however, produced inconclusive results. During the stimulation of the fornix, patients exhibited increased glucose metabolism at 6 months but not at 12 months [75]. Also, there was no significant improvement in clinical outcome measures between DBS ON and OFF groups. The authors concluded that the stimulation parameters applied to AD patients were not disease-specific and chosen based on parameters commonly used for DBS at other brain targets such as for Parkinson’s disease and tremor [75]. Moreover, the authors lacked an immediate clinical outcome for adjusting stimulation parameters, such as reduction of tremor that can be observed in DBS for Parkinson’s disease. In this case animal experiments might have been meaningful to define mechanisms of action of various stimulation paradigms. In particular, researchers should investigate whether current treatment paradigms (based on chronic stimulation regimes used in Parkinson’s patients) are necessarily the best approach to attempting to treat AD with DBS in humans. Other stimulation parameters, such as frequency, amplitude, and second-level patterning such as burst or pulse-train delivery may also affect outcomes and will require further investigation and optimization.

As directional leads and technological advancements improve, it would be meaningful to see whether stimulation parameters and sites (pre- or post-commissural fornix) can be tailored for the different indications. In addition, fornix DBS has only been performed so far in an open-loop manner in which stimulation is delivered continuously regardless of the physiological signals. However, it has been hypothesized that the timing and rhythmicity of neuromodulation may be crucial for functional activation of memory circuits that lead to long-term effectiveness [87, 88]. It has been shown in mice that DBS can enhance encoding and retrieval functions through theta phase-specific manipulation of the hippocampus [89] because they encompass different neurophysiological phenomena [90]. Likewise, another study has reported that patterned electrical stimulation of the fimbria–fornix increased theta-gamma co-modulation in amnestic rats and partially rescued memory performance during the water maze [91]. Interestingly, synaptic correlates of memory, such as long-term potentiation (LTP), have been shown to be sensitive to precisely timed electrical stimulation in behaving rats [92].

In the history of DBS, animal experiments have played a major role. Portraying the development of DBS for Parkinson’s disease, for example, Alim-Louis Benabid discovered that high-frequency stimulation of the thalamus was able to alleviate tremor [93]. A few years later, experimental studies on parkinsonian MPTP (1-methyl-4-phenyl-1,2,3,6-tetrahydropyridin) monkeys enabled the validation of the STN target [94]. Following these results, in 1993 a team from Bordeaux [95] showed the efficacy of high-frequency electric stimulation in the STN in two MPTP monkeys. This was a turning point in the application of DBS to Parkinson’s disease.

Yet, DBS was not initially tested on animals in all indications. For certain indications such as, obsessive–compulsive disorder (OCD) and depression, experiments on humans preceded animal experimentation [96]. This might be because animal models in psychiatric disorders are suboptimal and have limited face, construct, and predictive validity. It appears, that there is also no consensus about the necessary prior use of animal experimentation as is the case for drug marketing. Unfortunately, existing research methodologies, generally derived from drug trials, are often ill suited to invasive device trials due to a number of factors such as inappropriate study methods or resources available for trial design and subject follow up [97].

In summary, as research progresses a number of important issues will need to be addressed. First, new discoveries that contribute to the understanding of the molecular pathogenesis of AD and its relations are crucial as they allow for the greater development of tailored DBS. Second, applications of DBS in psychiatric disorders have been modeled after those used in movement disorders and might need modification accordingly. Therefore, the effects of unilateral versus bilateral stimulation as well as various stimulation parameters should be carefully considered and tested. Third, interpretation of animal studies should be taken with caution, as models of disease for psychiatric disorders are naturally imperfect.

## Conclusion

In the past 2 decades great advances in fornix DBS in both human patients and rodent models have led to multiple potential therapeutic methods for the treatment of brain diseases. As reviewed above, using different stimulation parameters in the fornix has shown therapeutic promise in both human patients and rodent models of brain diseases such as AD, RTT, TBI, and TLE. Researchers indicated that fornix DBS can be a feasible and safe approach.

Nevertheless, it is still unclear which stimulation patterns are most optimal within treatment methods of fornix DBS. These have typically been selected by experience based on a transcendental knowledge of neuroanatomy and clinical cases with DBS in other brain diseases. Therapeutic fornix DBS research is still in a period of infancy because of the inherent complexities within diverse disease processes, the challenging progression of preclinical models, and because of heterogeneous symptoms within patients. To propel future studies of fornix DBS forward, research needs to strengthen animal models, progress the refinement of patient selection, and continue to explore different stimulation parameters.
